# Paternity assignment in the polyploid *Acipenser dabryanus* based on a novel microsatellite marker system

**DOI:** 10.1371/journal.pone.0185280

**Published:** 2017-09-27

**Authors:** Ya Liu, Yeyu Chen, Quan Gong, Jiansheng Lai, Jun Du, Xiaochuan Deng

**Affiliations:** The Fishery Institute of the Sichuan Academy of Agricultural Sciences, Chengdu, China; SOUTHWEST UNIVERSITY, CHINA

## Abstract

*Acipenser dabryanus* is listed as a Critical Endangered species in the IUCN Red List and the first class protected animals in China. Fortunately, *A*. *dabryanus* specimens are being successfully bred in captivity for conservation. However, for effective *ex situ* conservation, we should be aware of the genetic diversity and the degree of relatedness of the individuals selected for breeding. In this study, we aimed at the development of novel and reliable microsatellites used for the genetic study of *A*. *dabryanus*. A total of 14,321 simple sequence repeats (SSRs) were detected by transcriptome sequencing and screening. We selected 20 novel and polymorphic microsatellites (non-dinucleotide) with good repeatability from the 100 tested loci for a subsequent genetic and paternity study. A set of captive broodstock (F1 stock, n = 43) and their offspring (F2 stock, n = 96) were used to examine the efficiency of the 20 SSRs for assigning parentage to offspring, with an allocation success of 91.7%. We also found that only a few families predominantly contributed to the progeny produced by the 43 breeders. In addition, mitochondrial DNA data showed that the captive broodstock (F1 individuals) had an excellent probability of the same lineage, implying that a high level of inbreeding may have occurred in these individuals. Our research provides useful information on genetic diversity and reproductive pattern of *A*. *dabryanus*, and the 20 SSRs developed in this study can be applied to the future breeding program to avoid inbreeding for this stock or other related species of Acipenseriformes.

## Introduction

*Acipenser dabryanus*, also called Dabry’s sturgeon, is an endangered species that is now extinct in reaches of the Yangtze River downstream of the Gezhouba Dam [1]. The wild population of *A*. *dabryanus* has drastically declined over the last two decades due to overfishing, habitat degradation and pollution [2]. Since 1982, only 150 specimens have been accidentally captured in the Yangtze River. No captures have been recorded below the Gezhouba Dam since 1995, and natural recruitment has not been documented in the upper Yangtze River since the 2000s [1, 3–5]. Consequently, *A*. *dabryanus* is listed as a Critical Endangered species on the International Union for Conservation of Nature and Natural Resources (IUCN) Red List [6], is a first class protected animal in China [7], and is considered a protected animal in Appendix II of the Convention on International Trade in Endangered Species of Wild Fauna and Flora (CITES) [1]. Thus, captive breeding of *A*. *dabryanus* and the release of captive-bred individuals into the wild may be the last attempt to ensure their survival and sustainability.

With *ex situ* breeding, one can obtain the complete genetic information for all populations and thus retain and transmit the optimal genetic diversity to the future generations of captive breeders [8]. In this context, information regarding a breeder’s pedigree is an important component in effective captive breeding. In fact, every *ex situ* population should be investigated genetically to determine whether it is a representative sample of the available genetic diversity, and the inbreeding level of the individuals selected for broodstock should be estimated. It is well established that mating between close relatives often leads to decreased fitness and that inbred individuals suffer from reduced viability and fecundity [9]. Therefore, a paternity analysis must be implemented in captive breeding to control and minimize the rate of mating with kin (mate choice) and to avoid inbreeding.

Our breeding program for *A*. *dabryanus* was initiated in 2012. However, the program has been conducted without any genetic information regarding F1 breeders or F2 offspring until now. No information is available for our F1 breeders regarding how many or which F0 wild parent pairs were used to produce these F1 animals; consequently, the relatedness of individuals is unknown. In addition, due to the limited space, we cannot disperse every offspring of a single parent pair into an independent pool, and therefore, a mixed culture is unavoidable when propagating *A*. *dabryanus*. Thus, we should establish a universal method for parentage analysis of the cultivated populations to maintain pedigree information.

Microsatellite makers (simple sequence repeats–SSRs) have become the most powerful genetic tools for determining parentage in aquaculture broodstocks because of their high variability. Their particular suitability stems from the combination of high variability with co-dominance [10]. Unfortunately, most methods developed for paternity assessment are only available for diploid organisms. Microsatellite analysis and a comparison of DNA content show that the ploidy in the order Acipenseriformes varies from 2N to 8N, with most species appearing to be 4N [11–14]. Thus, alternative methods are necessary for the paternity analysis of sturgeons and other polyploid species. The most common solution is to convert a polyploid genotype to a pseudodiploid genotype so that the numerous methods developed for diploids become available [15, 16].

So far, most of microsatellite markers developed for *A*. *dabryanus* are dinucleotide repeats [17–19]. Dinucleotide microsatellites often suffer from polymerase slippage [20, 21] which are difficult to score, especially in polyploid species. Generally, non-dinucleotide repeats are the optimal choice for paternity analysis because they tend to stutter less and are much more accurate and reliable. The number of microsatellite markers needed to obtain reliable results in a paternity test will depend upon the genetic variability of the species studied. When the genetic diversity is lower, a relatively larger number of microsatellites is needed [22]. However, captive breeding programs usually focus on species that have experienced population declines and have low genetic diversity. Therefore, in this study, we wanted to develop sufficient non-dinucleotide microsatellites with strong stability and very low genotyping error rates that could be widely used for the genetic management of *A*. *dabryanus* and might also be applicable to other sturgeons.

Traditionally, microsatellite loci have been isolated from partial genomic libraries of the species of interest by screening several thousand clones via colony hybridization with probes that contain repeats [23] which can be extremely tedious and inefficient for a species with a low microsatellite frequency. To increase the number of loci and to reduce the time invested in microsatellite isolation, we applied a high-throughput transcriptome sequencing strategy in our study.

In this study, we aim at developing sufficient, stable microsatellite markers for *A*. *dabryanus* by transcriptome screening. A large number of different types of repeat motifs of SSRs were identified. The 20 novel microsatellite loci screened from the contigs were suitable for a subsequent genetic study and paternity analysis of the captive stock and their progeny. To study the genetic diversity of broodstock and their progeny, the d-loop region in the mitochondrial DNA (mtDNA) of each individual was also analyzed. These results will help to increase our understanding of the genetic information regarding and the relatedness of captive *A*. *dabryanus*. Moreover, we established a reliable microsatellite-based method to conduct a paternity test in this polyploid species, which will further facilitate efficient domestication programs for *A*. *dabryanus* or other Acipenseriformes species.

## Materials and methods

### Ethics statement

All fish handling and experimental procedures were approved by the Animal Care and Use Committee of the Fishery Institute of the Sichuan Academy of Agricultural Sciences (20151212001a), and all animal collection and use protocols were carried out in accordance with the guidelines and regulations for the care and use of laboratory animals at the Fishery Institute of the Sichuan Academy of Agricultural Sciences.

### Sample collection

To obtain enough non-dinucleotide markers for transcriptome sequencing, two *A*. *dabryanus* specimens (8-month and 2-year) were sampled separately at the Fishery Institute of the Sichuan Academy of Agricultural Sciences. After anaesthetization with 0.05% MS-222 (Sigma, USA), gonad tissues were collected and immediately flash-frozen in liquid nitrogen and were then transferred to an ultralow freezer at −80°C until RNA extraction could be performed.

For the paternity test, 43 individuals (18 males and 25 females) transferred from the Yangtze River to the Fishery Institute of the Sichuan Academy of Agricultural Sciences (Chengdu, Sichuan, China) were treated as the F1 stock in this study. The 43 individuals were progeny of the wild sturgeon (F0). They were once released with tags when they were fry and were about 2-year-old when caught in Yangtze River and raised in captivity to reach maturation. Accordingly, the offspring reproduced by the breeders were considered as the F2 stock. To increase fertility rate, we usually use eggs of one female inseminated with milt from several males and the number of males used is variable according to the amount of the milt. We analyzed a total of 96 F2 individuals, containing 29 offspring obtained in 2013, 43 offspring from 2014 and 24 offspring from 2015. The 43 individuals were all the alive F1 sturgeon while the 96 individuals represented a large subset of the whole progeny.

Genomic DNA was extracted from a fin-clip (30–50 mg) using the cetyltrimethylammonium bromide (CTAB) method and was stored at -20°C.

### RNA isolation, library construction and Illumina sequencing

Total RNA isolation was performed using a TRIzol kit (Invitrogen, Carlsbad, CA) according to the manufacturer’s instructions. RNA degradation and contamination were monitored on a 1% agarose gel. RNA purity was checked using a NanoPhotometer^®^ spectrophotometer (IMPLEN, CA, USA). The RNA concentration was measured using a Qubit^®^ RNA Assay Kit in a Qubit^®^ 2.0 Fluorometer (Life Technologies, CA, USA). The RNA integrity was assessed using an RNA Nano 6000 Assay Kit for the Agilent Bioanalyzer 2100 system (Agilent Technologies, CA, USA). Sequencing libraries were generated using the NEBNext^®^ Ultra^TM^ Directional RNA Library Prep Kit for Illumina^®^ (NEB, USA) following the manufacturer’s recommendations. Briefly, enriched mRNA was used for first-strand cDNA synthesis, followed by second-strand cDNA synthesis. Final cDNA libraries were obtained by PCR amplification and purification and were paired-end sequenced with the Illumina HiSeq ^2000^.

### De novo assembly of sequencing reads

Raw data (raw reads) in fastq format were initially processed through in-house Perl scripts. Clean data (i.e., clean reads) were obtained by removing reads containing adapters, reads containing poly-N and low-quality reads from the raw data. In this step, all raw reads with mis-sequenced nucleotides (reads were N) larger than 10% were discarded, and reads with more than 50% of bases having Q-values≤20 were filtered out. All clean reads were assembled into the two libraries with the short read assembling program Trinity [24]. Sequences assembled in Trinity were contigs, which were grouped together to conduct the final assembly for the subsequent analysis of microsatellite loci. The transcriptomes were named T1 (the transcriptome for the 8-month-old sturgeon) and T2 (the transcriptome for the 2-year-old sturgeon).

### Identification and development of microsatellite markers

The microsatellite regions from the sequence data were identified using the program QDD 3 [25]. To assess the distribution and characterization of the microsatellite abundance, di-, tri-, tetra-, penta-, and hexanucleotide motifs were screened from the scaffolds. In the search for an SSR standard, we defined an SSR as a dinucleotide ≥14 bases, a trinucleotide ≥18 bases, a tetranucleotide ≥24 bases, a pentanucleotide ≥30 bases, and a hexanucleotide ≥36 bases. To eliminate redundancy, the similarity of sequences containing the microsatellite regions was analyzed via all-against-all BLAST searching. Further criteria of microsatellite identification included: (1) repeats should not be dinucleotide repeats; (2) microsatellites should not be in published repeat sequences; (3) the number of repeats should be in the range of 6–22; and (4) the flanking sequences of microsatellites must be long enough to design a primer (i.e., more than 20 bp). Primer pairs were designed using Primer 3.0 software [26] to meet the following restrictions: the target amplification size range was 120–500 bp, the primer annealing temperature was restricted to 55–65°C and the primer length was 20–27 bp. The presence of structures such as hairpins or short repeat motifs was also considered while designing the primers. We termed such loci detected with PCR primers as potential microsatellite markers. We then tested these primers for reproducible amplification in three *A*. *dabryanus* samples under the standard PCR conditions, with annealing temperatures altered according to the primer sequence.

### Polymorphism microsatellite isolation

The tissue DNA from 43 captive *A*. *dabryanus* was used to evaluate the capacity of the primer pairs to amplify polymorphic bands, the primers were labeled with one of three fluorescent dyes (FAM, TAMRA or HEX) (Invitrogen, Shanghai, China). PCR amplifications were performed in a 20-μL reaction volume containing 50–100 ng of genomic DNA, 0.5 μM of each primer, 10 μL 2× Taq PCR MasterMix (0.1 unit Taq polymerase/μL, 0.5 mM dNTP each, 20 mM Tris-HCl [pH 8.3], 100 mM KCl, and 3 mM MgCl_2_; Tiangen Biotech, Beijing, China). PCR amplifications were conducted under the following conditions: an initial denaturation for 4 min at 95°C; followed by 32 cycles at 94°C for 30 s, at the annealing temperature for each specific primer (optimized for each locus) for 30 s, and at 72°C for 30 s; and a final extension step at 72°C for 8 min. For genotyping, the PCR amplification products were separated via capillary electrophoresis using a denaturing acrylamide gel matrix on an ABI 3730XL (Applied Biosystems) using a GeneScan LIZ 500 size standard. Alleles were detected using Genemarker software, and the markers that formed stutter peaks were then manually excluded.

### Microsatellite data transmission

The markers that showed high polymorphism and good repeatability were used to genotype all of the 43 F1 and 96 F2 individuals. We used the classic band-sharing approach as proposed by Congiu *et al*., which considered the presence/absence of bands and disregarded the number of alleles present in each individual [27]. Each microsatellite band is scored as an individual dominant locus as proposed by Rodzen [28]. Phenotypes were converted to a series of dominant loci, where the value for the genotype should be either 1 (dominant phenotype, presence of a band) or 2 (recessive phenotype, absence of a band).

### Microsatellite data analysis

Microsatellite polymorphism analyses, including the number of alleles (Na), the expected heterozygosity (H_E_), and the Shannon-Wiener Diversity Index (*H’*), were performed using ATetra 1.2 software.

For the parentage analysis, we used COLONY 2.0, a software package that can be applied to polyploid species with slight modification of the data [29, 30], to assign 96 F2 individuals to unknown candidate parents (i.e., the F1 stocks containing 43 individuals) based on the microsatellite loci developed in this study. To infer the parentage, the software implemented a full-pedigree likelihood approach. The approach considers the likelihood of the entire pedigree structure and jointly infers parentage and sibships. The data consisted of genotypes of the candidate fathers (CFS), candidate mothers (CMS) and offspring (OFS). COLONY was run with the default parameters and set for a medium run and a full-likelihood analysis, assuming with inbreeding and clone. Only parental assignments with greater than 95% probabilities were considered.

Prior to the paternity test, the accuracy of the method was tested on 20 F2 individuals (two groups of full-sibs) of known pedigree to evaluate the efficiency of the converted data and the application of COLONY in a polyploid species. Using the 43 individuals of the F1 group as putative founders, all 20 F2 individuals were allocated to the correct parent pair with 100% accuracy.

### Mitochondrial DNA analysis

The entire mitochondrial control region, including partial sequences of the flanking tRNA genes, was amplified and sequenced for all F1 and F2 individuals. The primers used matched proline (F: 5’-GCCCTAGTAGCTTAGACATCAAAG-3’) and phenylalanine tRNAs (R: 5’-GTGCGTGCCTGATACCTGC-3’), respectively. PCR amplification was performed in a 50-μL reaction volume containing 100 ng of genomic DNA, 1 μM of each primer, and 25 μL 2× Taq PCR MasterMix. PCR amplification was conducted under the following conditions: an initial denaturation for 4 min at 95°C; followed by 32 cycles at 94°C for 30 s, at the annealing temperature 54°C for 30 s, and at 72°C for 1 min; and a final extension step at 72°C for 8 min. Sequencing reactions were performed using an ABI Prism3730XL automatic sequencer. mtDNA sequences were aligned using ClustalW in MEGA6.

## Results

### Overview of the assembly results

Two libraries from different individuals were established and sequenced. In total, 44,097,314 raw reads were obtained from the high-throughput sequencing data of the *A*. *dabryanus* transcriptome (S1 Table). The GC percentages were 48.58% from T1 and 49.16% from T2. The Trinity program was used for the assembly of the RNA sequencing (RNA-Seq) short reads, and a total of 908,226 contigs were obtained, ranging from 200 to 16,697 bp (S2 Table). The length distribution of the transcripts is shown in S1 Fig. Of 908,226 contigs, 164,809 (18.14%) were more than 1,000 bp long. The respective N50 and mean lengths of the contigs were 1,084 and 682 bp in the T1 transcriptome and 1,110 and 691 bp in the T2 transcriptome.

### SSR frequency and distribution in the *A*. *dabryanus* transcriptome

**Fig 1:**
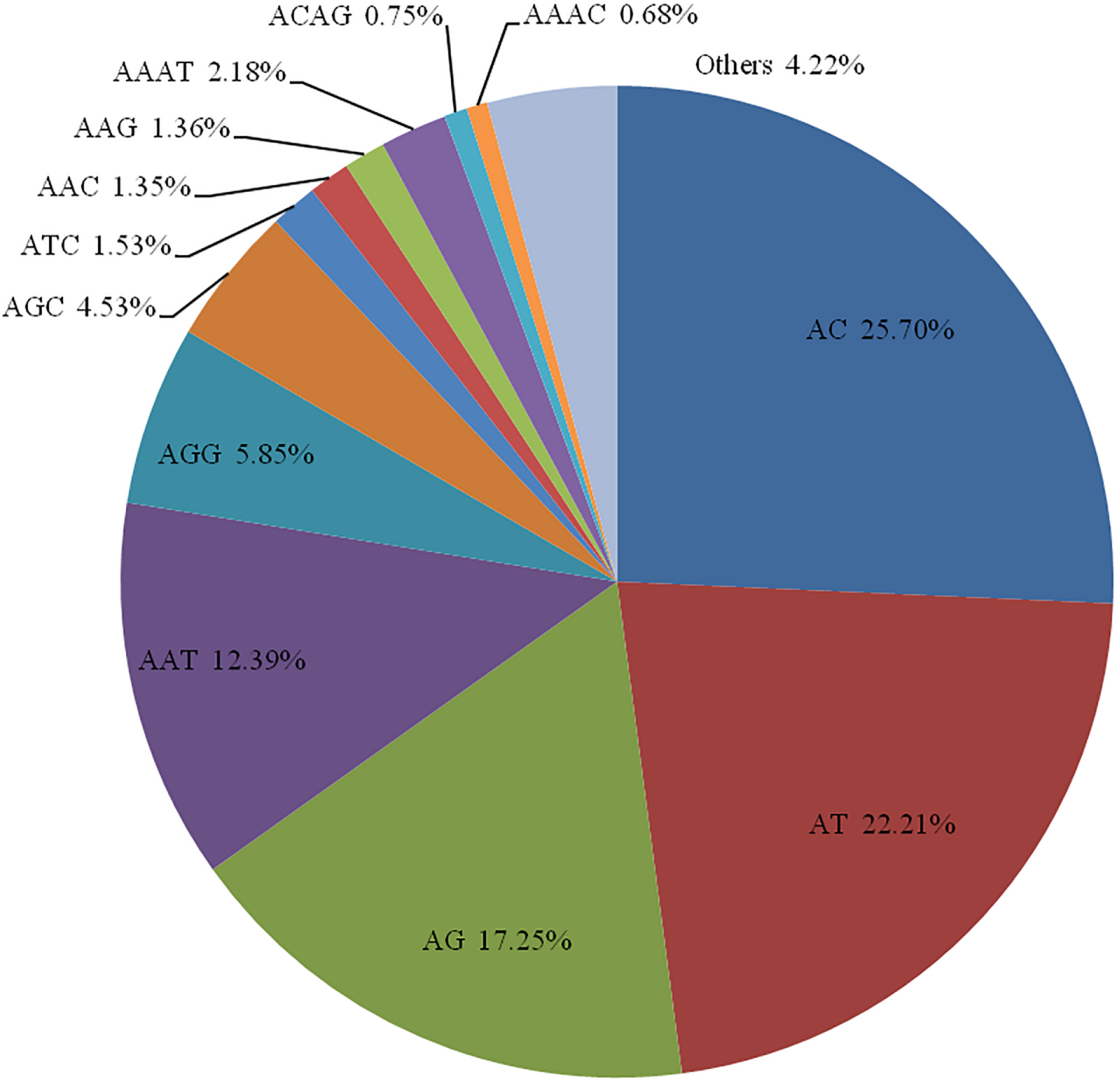
Frequency of microsatellite motif categories in the *A*. *dabryanus* transcriptome. The 12 most frequent microsatellite motifs are shown in divisions.

A total of 14,321 SSRs were identified from the transcriptomes of *A*. *dabryanus* (S3A and S3B Table). Disregarding mononucleotides, dinucleotides were the most abundant category, accounting for 65.20% of all SSRs, followed by trinucleotides (28.34%) and tetranucleotides (5.98%), as shown in S2 Fig. In contrast, penta- and hexanucleotides were less abundant. Among all dinucleotide repeat categories, (AC)n and (AT)n were the two most frequent microsatellite motifs (Fig 1). Similar to other fishes [31], (GC)n repeats were extremely rare in *A*. *dabryanus*. Over 80% of the trinucleotide types were (AAT)n, (AGG)n and (AGC)n in the *A*. *dabryanus* transcriptomes. The most abundant tetranucleotide motifs were (AAAT)n, (ACAG)n and (AAAC)n, comprising approximately 36.49%, 12.63% and 11.46% of the total number of microsatellites of this repeat category, respectively.

### Development of the microsatellite markers

We chose 100 loci and designed 100 pairs of primers for amplification, with target product sizes of approximately 100–400 bp, respectively. After amplification, 51 loci that showed a single band of the expected size were further considered. Loci that failed to provide signals or lacked polymorphisms were disregarded. A total of 31 novel microsatellite loci showed polymorphisms for *A*. *dabryanus*. We then further screened the loci that showed good polymorphisms and clear fluorescent peak patterns. Finally, we selected 20 microsatellites for a subsequent paternity analysis (designated as the 20 ADX system). The number of alleles per locus ranged from 2 to 10, with an average of 5.3. The mean expected heterozygosity (H_E_) and the Shannon-Wiener Diversity Index (*H’*) per locus ranged from 0.6341 to 0.8569 and from 0.9567 to 1.9863, respectively (Table 1).

**Table 1:** Characteristics of the novel microsatellite marker system of *Acipenser dabryanus*.

Locus	Repeat motif	Primer(5’-3’)	Ta (°C)	Size (bp)	Na	H_E_	*H’*	Fluorescent dyes	GenBank Accession no.
ADX11	(AGC)7	F:AAACTTACTGAGAACCTGGAGC R:GCTAGAATTGCTGCCACTAGAA	55	150–156	3	0.7237	1.3251	FAM	KY364815
ADX21	(ATC)19	F:AACTTCATCATGCTGGAA R:GATTTGACTGCTTTGTGG	54	116–154	8	0.8569	1.9863	FAM	KY364816
ADX29	(ACCCTC)6	F:CCTCGTCACCACATACATCA R:TTATTAAGGGACCCTCCTAG	60	418–452	7	0.7830	1.3898	TAMRA	KY364817
ADX33	(ACAGG)11	F:GTGGGCAAAGACCTCAGACA R:CTTGGGACAGTGGCAACGAC	64	367–442	10	-	-	TAMRA	KY364818
ADX37	(CTTT)13	F:TGTGGAATTTCTATAACCTTGA R:TATAACATGCAAAAAGATTGGT	59	202–219	2	0.6433	1.0595	HEX	KY364819
ADX38	(GATT)12	F:GTTATTGACCTCACAGAAGACG R:TTCTAGGTGCTGCTAAAGTTTC	59	123–144	6	-	-	FAM	KY364820
ADX40	(AAAC)11	F:CCTCAGTTTGGGAGTATAGGAGA R:AGTTTACACAGGCTTGATGTTGT	63	141–153	4	0.7501	1.3111	FAM	KY364821
ADX42	(GTGTT)6	F:CTCCTGTGGGTTTTGCCTG R:CTTCCCTGTGTTCGTGTGA	61	93–115	5	0.7367	1.2185	FAM	KY364822
ADX52	(CGGGG)7	F:AAGTACAAACAGAGAAGCAGCG R:AAATAAACCTGCAGTGACCG	61	180–195	4	0.6729	0.9567	HEX	KY364823
ADX55	(CCT)9	F:ACCATGCACTGCTTTACTACAA R:AAAGAACAGTTTGAAATCATTCG	63	176–203	7	-	-	HEX	KY364824
ADX58	(CCAGTT)7	F:ATGCTCTTGATTTATAGAAGGGT R:ACAAACACAGAAACAGAACGGAA	57	141–164	3	0.6341	1.0057	FAM	KY364825
ADX59	(CCTCT)6	F:GCACTCTTGAGGTGTATCAGTT R:GAACCGTGTCTGTGGAACCAG	61	203–231	4	0.6427	1.0650	HEX	KY364826
ADX60	(GGAA)8	F:AGCTGGGGTCGTACATTTTTAA R:GGAGGGTCACCTTCCTTTCTTT	61	130–159	6	0.7927	1.2871	FAM	KY364827
ADX61	(TCTG)8	F:TGACCAAACCAAGGAACTACA R:AAAGGGTTTGCAATGCTGCC	62	207–219	3	0.6515	1.0668	HEX	KY364828
ADX66	(AGCCG)7	F:CCGCTTCCTGAAACTAGAGAG R:TCTAAACAGAACGGTATCCCTG	59	238–270	6	-	-	TAMRA	KY364829
ADX70	(TTA)22	F:AAATTTGATCCTCTGTCATGGA R:GAGAATGTCCAACTTCAATACCTT	62	294–324	7	0.8313	1.1143	TAMRA	KY364830
ADX72	(GGGGT)9	F:GCCTGGGAGAGAAGTGTTGA R:GGAGGGGAGAGTGGGAGATA	59	219–234	4	0.7021	1.1020	HEX	KY364831
ADX74	(GAA)14	F:AATGAAGGGACTGCGTAAT R:CTTTCCAGGGACAGATGTG	60	260–283	7	-	-	TAMRA	KY364832
ADX75	(AATAG)6	F:AATATAGAGAAATGCCCATCCCT R:AGCCACATTGACTTGGAACTAAC	59	385–405	4	0.7458	1.4019	TAMRA	KY364833
ADX80	(ATG)14	F:CTCTCATTTCTCTTCTGTCGCTA R:AAAGTCTTCCTCAATTTACCCTC	48	186–220	6	0.7805	1.3956	HEX	KY364834

“-” means the loci with higher than 4 alleles that could not be conducted in ATetra or other software to estimate the H_E_ or *H’*.

### The ploidy of *A*. *dabryanus*

Only one study in the literature implied that *A*. *dabryanus* was octoploid by comparing its DNA content with that of the American paddlefish (*Polyodon spathula*) [14]. In the present study, among all of the individuals we studied, 15 out of 20 loci showed no more than 4 alleles. However, the remaining 5 loci (ADX33, ADX38, ADX55, ADX66 and ADX74) displayed more than 4 bands in most individuals in either the F1 or F2 stock. Allelic band patterns indicating the ploidy of *A*. *dabryanus* at the 20 microsatellite loci are shown in Table 2. Among the 43 F1 individuals, the percentage of individuals who showed more than 4 bands were 95.3%, 67.4%, 60.5%, 28% and 95.3% at the loci ADX33, ADX38, ADX55, ADX66 and ADX74, respectively. No more than 8 alleles were found for each amplified locus of all 43 individuals.

**Table 2:** The number of alleles calculated in 20 microsatellite loci in 43 parent individuals.

Locus	Least number of alleles in each individual	Greatest number of alleles in each individual	Percentage of individuals higher than tetrasomic(%)	Inferred ploidy
ADX11	2	3	0	4n
ADX21	3	4	0	4n
ADX29	2	4	0	4n
ADX33	4	7	95.3	>4n
ADX37	1	2	0	2n
ADX38	3	6	67.4	>4n
ADX40	3	4	0	4n
ADX42	2	4	0	4n
ADX52	1	4	0	4n
ADX55	3	7	60.5	>4n
ADX58	1	3	0	4n
ADX59	1	3	0	4n
ADX60	2	4	0	4n
ADX61	2	3	0	4n
ADX66	2	5	28	>4n
ADX70	2	4	0	4n
ADX72	1	3	0	4n
ADX74	4	7	95.3	>4n
ADX75	2	4	0	4n
ADX80	2	4	0	4n

### Mitochondrial DNA sequence variation

A total of 727 bp of the mitochondrial DNA control region were analyzed after removing the partial sequences of the flanking tRNA genes. To our surprise, all 43 parental stock specimens and 96 offspring F2 stock shared the same haplotype. No changes in the haplotype and nucleotide diversity were found in any individual.

### Parental allocation

**Fig 2:**
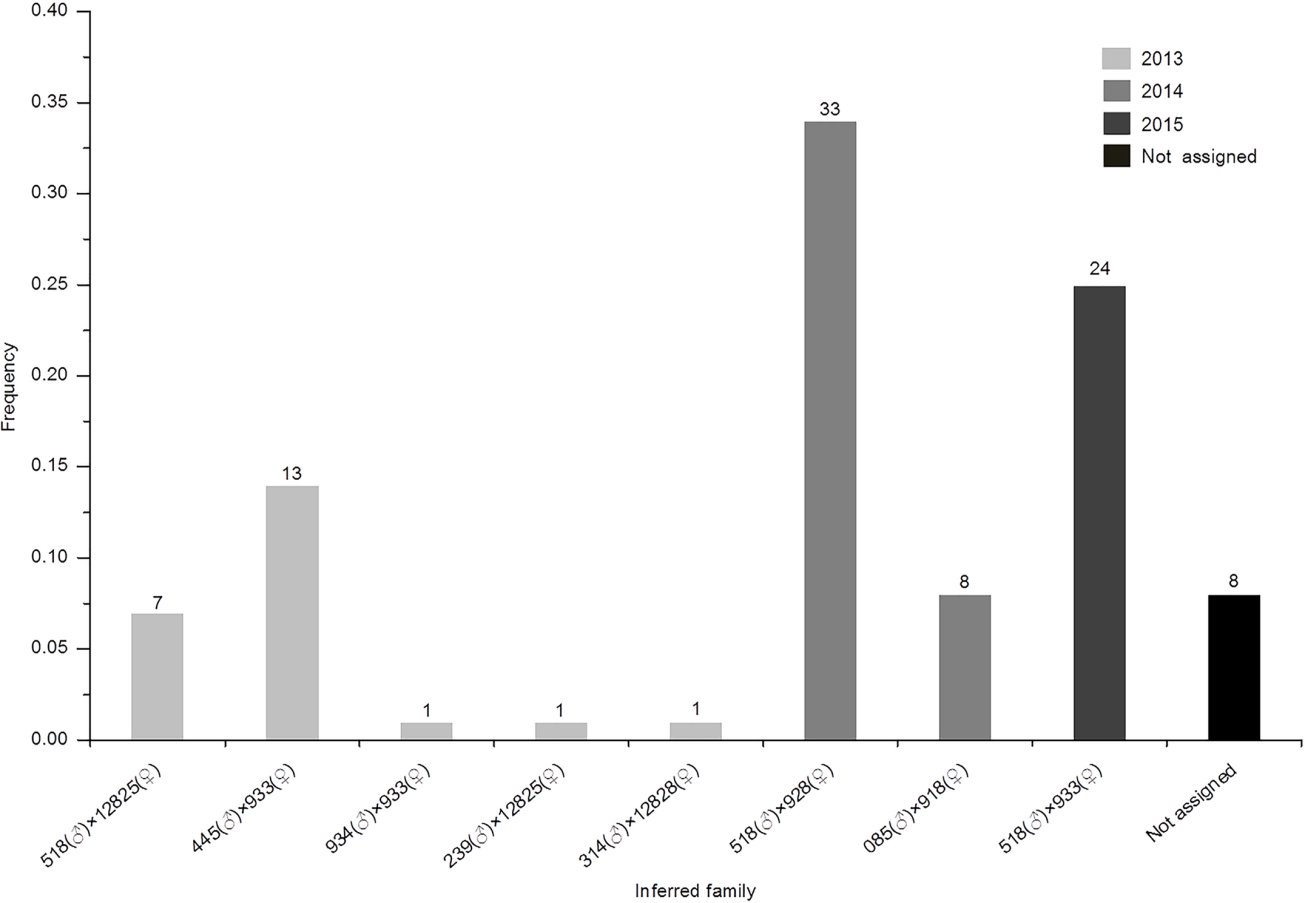
Proportion of the offspring assigned to each mating pair inferred from the parentage analysis. The number of offspring in each cohort is shown above the bar.

Of the 96 offspring analyzed from three different spawning groups (29 from the year 2013, 43 from the year 2014 and 24 from the year 2015), 70 were assigned to just three families (33, 24 and 13 for families 518♂×928♀, 518♂×933♀ and 445♂×933♀, respectively). The remaining individuals were assigned to five other full-sib families, each of which consisted of one to eight individuals, detailed results of the parental allocation is shown in S4 Table. All 24 offspring from 2015 were assigned to family 518×933, and 33 of 43 individual offspring from 2014 came from family 518×928, while offspring from 2013 were allocated to more diverse families. Eleven of the 43 brooders participated in the matings, contributing 66.7%(518♂), 13.5%(445♂), 8.3%(085♂), 1.0%(934♂), 1.0%(239♂), 1.0%(314♂), 39.6%(933♀), 34.3%(928♀), 8.3%(918♀), 8.3%(12825♀) and 1%(12828♀) in the cohort respectively. It should be noticed that only three individuals (518, 928 and 933) contributed up to 88.6% of the individuals (Fig 2). For paternity, 8 out of the offspring were not assigned to their father, while for maternity, 5 out of 96 F2 individuals were not compatible with any possible female parent, which corresponds to an allocation success of 91.7%. The genotype result of the 43 captive *A*. *dabryanus* and 96 offspring based on 20 microsatellites are shown in S4 Table. No multiple allocations were observed, confirming the high resolving power of the markers.

## Discussion

### Transcriptome assembly

Dabry’s sturgeon is one of the world’s threatened species and urgently needs protection. However, limited genetic information for Dabry’s sturgeon has hindered studies at the molecular level. This study was the first attempt of an investigation of the *A*. *dabryanus* transcriptome. A transcriptome-based strategy for obtaining molecular markers has better performance than traditional PCR-based methods and can be applied to species for which genome sequence information is lacking [32]. Though transcriptome-derived SSRs should be subjected to stronger selection pressure compared to genomic SSR as they are associated with transcribed [33], lots of comparative analyses suggest that only a very small percentage of all genes are experiencing positive selection [34, 35]. What’s more, single-generation applications such as parentage analysis is less sensitive to the effects of selection and the effects of selection can be minimized by increasing the number of markers used [36]. Therefore, in present study, we aim at developing sufficient markers from transcriptome sequencing to increase the reliability of the paternity test results. The transcriptome-derived SSR markers often showed a high transferability rate across the genus [37], so the markers developed in this study may also be applicable in genetic study of other sturgeons.

In this study, we used RNA-seq technology to identify a large number of SSRs that would assist subsequent genetic research on the endangered species *A*. *dabryanus*. In total, 908,226 contigs were assembled, with mean lengths of 682 and 691 bp for the T1 and T2 transcriptomes, respectively. The mean lengths of the contigs (S2 Table) were longer than those of the lake sturgeon (342 bp) [38], the Adriatic sturgeon (520 bp) [39] and the Russian sturgeon (584 bp) [40] and were slightly shorter than that of the Chinese sturgeon (706 bp) [31]. The contigs obtained were useful for SSR detection and future annotation with various protein databases for Dabry’s sturgeon.

### Distribution and organization of microsatellites in the *A*. *dabryanus*

In this study, we characterized the SSRs in the transcriptome sequencing assembly of Dabry’s sturgeon and analyzed their types and frequencies. Disregarding mononucleotides, most of the SSRs were di- and trinucleotides, accounting for up to 93.6% of all of the SSRs identified. In vertebrates, (AC)n is the most common dinucleotides motif followed by (AT)n. (G+C)-rich motifs (e.g., AGC, AGG) are the most common among trinucleotides in exons while (AAT)n dominate in introns [41]. The distribution of microsatellites in the Dabry’s sturgeon was generally in agreement with that in vertebrates. Due to the low frequencies of tetra-, penta- and hexanucleotides that displayed higher stability in the paternity analysis than shorter repeats, we constructed two separate libraries to obtain enough reliable markers of these types. The diverse motifs and the large pool of microsatellites discovered in this study provide the potential to greatly expand the limited microsatellite marker resources available for this vulnerable species, and the longer repeat (at least trinucleotides) microsatellite data will be helpful in developing SSR markers that could allow the establishment of a stable marker system for genetic studies. In this study, we finally selected 20 microsatellite loci that had at repeat motifs of at least three nucleotides with clear signals and high stability. These loci could be widely and effectively used in the subsequent paternity testing and genetic management of Dabry’s sturgeon.

### The ploidy level of *A*. *dabryanus*

Sturgeons provide an ideal taxonomic context for the examination of genome duplication events. Multiple ploidy exists among these fish. Different degrees of ploidy are the result of multiple and independent duplication events and allow sturgeon species to be divided into two groups (approximately 120 and 240 chromosomes), while the division of the species into two groups of either diploid/tetraploid or tetra/octoploid is unclear [42, 43]. Thus far, the ploidy of Dabry’s sturgeon remains controversial, and the exact chromosome number and karyotype for this species has not yet been documented in the literature. Zhang *et al*. inferred that Dabry’s sturgeon was octoploid by determining the genome size (i.e., from the somatic nuclear DNA content) using chicken erythrocyte DNA as a standard [14]. Que *et al*. and Zeng *et al*. indicated a tetraploid scale for Dabry’s sturgeon in a microsatellite study [18, 19]. In their research, no more than four alleles in each amplified locus were found among all of the individuals they studied. However, in this study, of the 20 loci we genotyped, 5 showed more than four alleles in majority of the individuals. There are two possible reasons for this phenomenon: i) the ploidy of Dabry’s sturgeon is larger than 4n; and ii) the 5 loci are within duplicated regions. Since there was no genomic information available for sturgeons, to address the second problem, we tried to use the sequence of these loci to blast in the database and find the corresponding sequence in other fish whose genomic information were available. Unfortunately, we can not map these loci. Therefore, further studies (e.g., chromosome and karyotype analysis) are required to ensure the ploidy level of Dabry’s sturgeon.

### Parental allocation success

In polyploid species, a phenotypic heterozygote may have several possible underlying genotypes with various numbers of copies of one or more alleles [30]. For example, in octoploids, the phenotypes AB, ABC and ABCD can have 7, 21 and 35 possible genotypes, respectively, which cannot be distinguished using current experimental approaches. The uncertainty of the genotype data makes it impossible to conduct a paternity test in polyploids using traditional methods that apply to diploid species. One solution is to convert the polyploid genotype to a pseudodiploid genotype and then can be analyzed using methods developed for diploid dominant markers. Thus far, empirical data have been used in several polyploid species to obtain accurate paternity assignments from converted data with standard methods developed for diploid species [15, 16, 28]. Because data transformation would inevitably cause a loss of information, and thus, a reduction in the accuracy of the analysis, we tested 20 F2 individuals (two groups of full-sibs) of known pedigree to evaluate the efficiency of converted data and the application of the software in the polyploids. Because we identified sufficient reliable SSR markers, all 20 F2 individuals were allocated to the correct parent pair with 100% accuracy using the 43 individuals of the F1 group as putative founders. We then used the 20 ADX microsatellite system to conduct a paternity test of the 96 offspring whose parents were unknown. The 20 ADX microsatellite system assigned the F2 progeny to the correct F1 parental pair with high efficiency. All positive allocations were unequivocal, with no ambiguities due to multiple allocations. In total, 8 out of the 96 offspring failed to compatible with any parental pair. Unfortunately, there were several Dabry’s sturgeons died since caught. In deed, we used four additional reported microsatellites spl101 [11], ds14 [19], ds16 [19] and Aox27 [11] to genotype the unassigned offsprings, they still failed to assign to any F1 individuals, which suggests that those parents might have died and were not included in our sampling. Our analysis showed a high allocation power for a paternity test for polyploids, which was likely due to the relatively high number of marker loci we used and their good stability when genotyped in all of the individuals.

### Mitochondrial variability

To assess the genetic variability of the F1 and F2 stock and to facilitate the paternity test, we analyzed the d-loop sequences of all the individuals in this study. Surprisingly, all the individuals shared only one haplotype, which may imply a dramatic loss of genetic diversity of the captive Dabry’s sturgeon and their progeny. At the microsatellite level, we also found that the number of alleles per locus ranged from 2 to 10 with an average of only 5.3. This loss of genetic variation is likely a consequence of relatedness, with F1 individuals being more related than the parental wild stock. Because wild parental specimens are impossible to obtain now, we are not able to demonstrate the actual relationships among the 43 F1 stocks. However, mtDNA and microsatellite data showed that all F1 individuals had a high probability of arising from the same lineage, implying that a high level of inbreeding may have occurred in these 43 individuals.

### The 20 ADX system

The primary purpose of the present study was to isolate sufficient stable and reliable microsatellite markers to construct a paternity testing system for polyploid Dabry’s sturgeon. Microsatellites suitable for this purpose are those that have multiple alleles and high heterozygosity. However, it should be emphasized that the populations used in this study were highly inbred and had much less genetic polymorphism and heterozygosity than a population with less or no inbreeding, increasing the difficulty of achieving the objective. Assigning a true parent in a group containing more highly related individuals is difficult because they share similar marker patterns. Therefore, a relatively larger number of microsatellites is needed in a highly inbred population to conduct a paternity analysis. Fortunately, the 20 ADX system developed in this study was highly efficient in a paternity test in the highly inbred population of Dabry’s sturgeon, implying that the 20 markers had a much higher allocation power in a population without inbreeding and may be useful in a parentage analysis for other sturgeons.

### The conservation concern

The parentage analysis in three different spawning episodes in the present study suggested that almost 80% of individuals in a cohort came from only three full-sib families and that only three brooders contributed nearly 90% of the offspring. Specifically, progeny from 2013, 2014 and 2015 came from four, three and one family, respectively. In other words, only a few families predominantly contributed to the progeny. Aquaculture stocks of several fish species that rely on a similar breeding strategy have encountered similar problems [44–47]. In our case, together with mtDNA and microsatellite data, a high level of inbreeding may have occurred in the Dabry’s sturgeon broodstock. The broodstock from the Yangtze River has a high probability of coming from a very limited parent pair. To protect Dabry’s sturgeon, a first class protected animal, it is urgent to increase its genetic diversity to help prevent damage from inbreeding, which can be achieved by introducing new breeders to increase the broodstock population size, genotyping selection candidates and composing broodstocks based on the restriction of molecular coancestry. The data developed in the present study can be applied to an introduction program with successful mate choice for this stock or other related Acipenseriformes species.

## Supporting information

S1 Table Statistics of sequencing quality of *Acipenser dabryanus* transcriptomes. (DOC)

S2 Table Summary of the *Acipenser dabryanus* transcriptomes assembly. (DOC)

S3 Table SSRs identified from the T1 transcriptome (S3A) and the T2 transcriptome (S3B) of *A. dabryanus*. (XLSX)

S4 Table Individual genotypes and results of parental allocation of the 43 captive *A*. *dabryanus* and 96 offspring based on 20 microsatellites. (XLSX)

S1 Fig Length distribution of contigs obtained from all of the libraries. The length statistic of the contigs from the *A*. *dabryanus* transcriptome.
(TIF)

S2 Fig Summary of SSR types in the *Acipenser dabryanus* transcriptome. (TIF)
