# Supplementary material for: Paternity assignment in the polyploid Acipenser dabryanus based on a novel microsatellite marker system
Source: PLoS One. 2017 Sep 27;12(9):e0185280. doi: 10.1371/journal.pone.0185280 (PMC5617196; doi:10.1371/journal.pone.0185280)
Supplement: S1 Table — (DOC) [file pone.0185280.s001.doc]

**Table 1. Statistics of sequencing quality of *Acipenser dabryanus* transcriptomes**

| Sample | Raw reads | Clean reads | Raw bases(G) | Clean bases(G) | Error(%) | Q20(%) | Q30(%) | GC(%) |
| --- | --- | --- | --- | --- | --- | --- | --- | --- |
| T1 | 20,740,200 | 20,297,830 | 6.21 | 6.07 | 0.03 | 95.60 | 91.10 | 48.58 |
| T2 | 23,357,114 | 22,852,943 | 6.99 | 6.84 | 0.03 | 95.61 | 91.09 | 49.16 |
