# Supplementary material for: Paternity assignment in the polyploid Acipenser dabryanus based on a novel microsatellite marker system
Source: PLoS One. 2017 Sep 27;12(9):e0185280. doi: 10.1371/journal.pone.0185280 (PMC5617196; doi:10.1371/journal.pone.0185280)
Supplement: S2 Table — (DOC) [file pone.0185280.s002.doc]

**Table 2. Summary of the *Acipenser dabryanus* transcriptomes assembly**

| Contigs statistic | T1 | T2 |
| --- | --- | --- |
| Total no. of contigs | 484,439 | 423,787 |
| Total no. of length | 330,627,700 | 292,780,512 |
| Average length of contigs | 682 | 691 |
| Max length | 16,697 | 15,581 |
| N50 length | 1,084 | 1,110 |
