# Supplementary figures and images for: Paternity assignment in the polyploid Acipenser dabryanus based on a novel microsatellite marker system
Source: PLoS One. 2017 Sep 27;12(9):e0185280. doi: 10.1371/journal.pone.0185280 (PMC5617196; doi:10.1371/journal.pone.0185280)

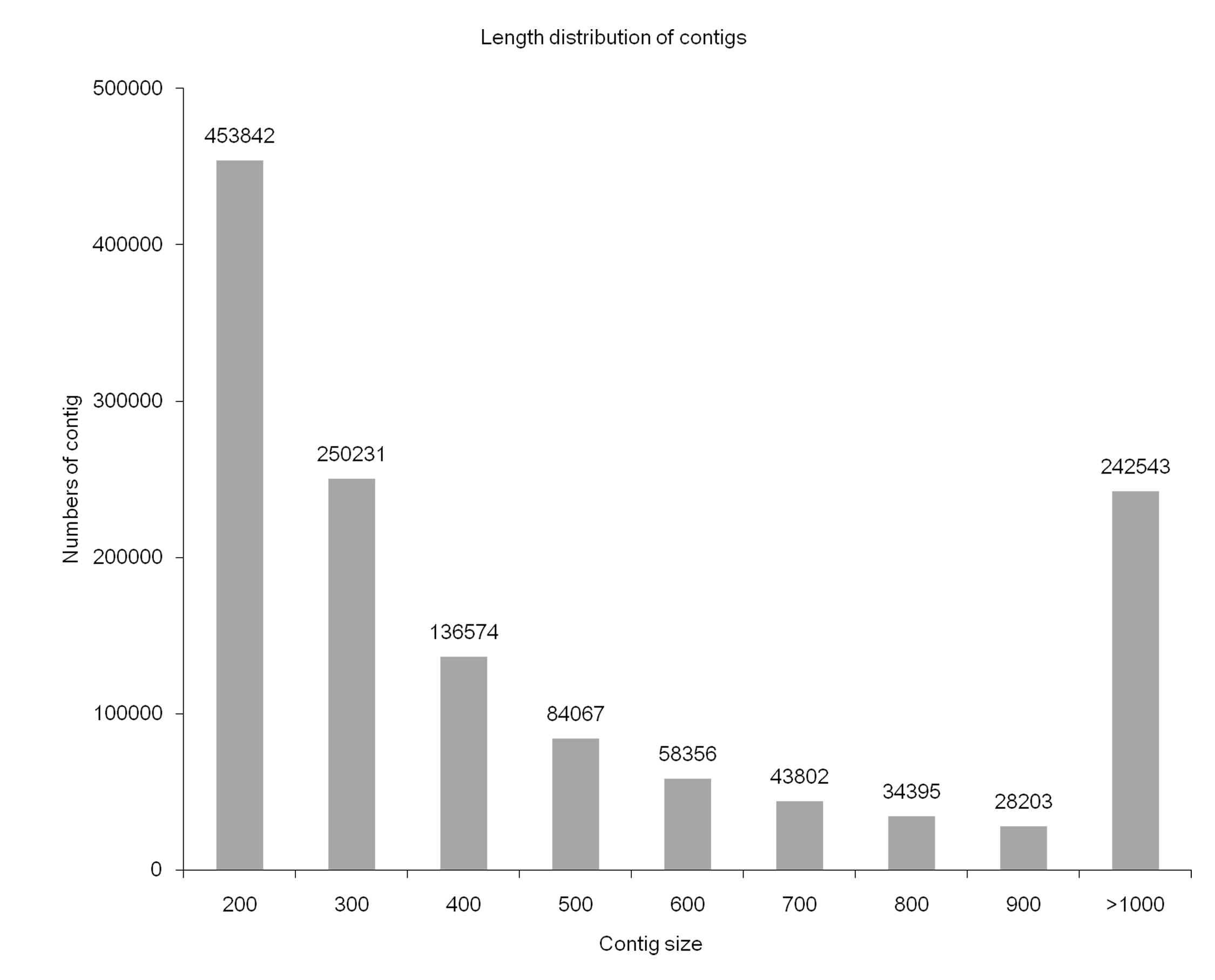

Supplement: S1 Fig — The length statistic of the contigs from the A. dabryanus transcriptome. (TIF) [file pone.0185280.s005.tif]

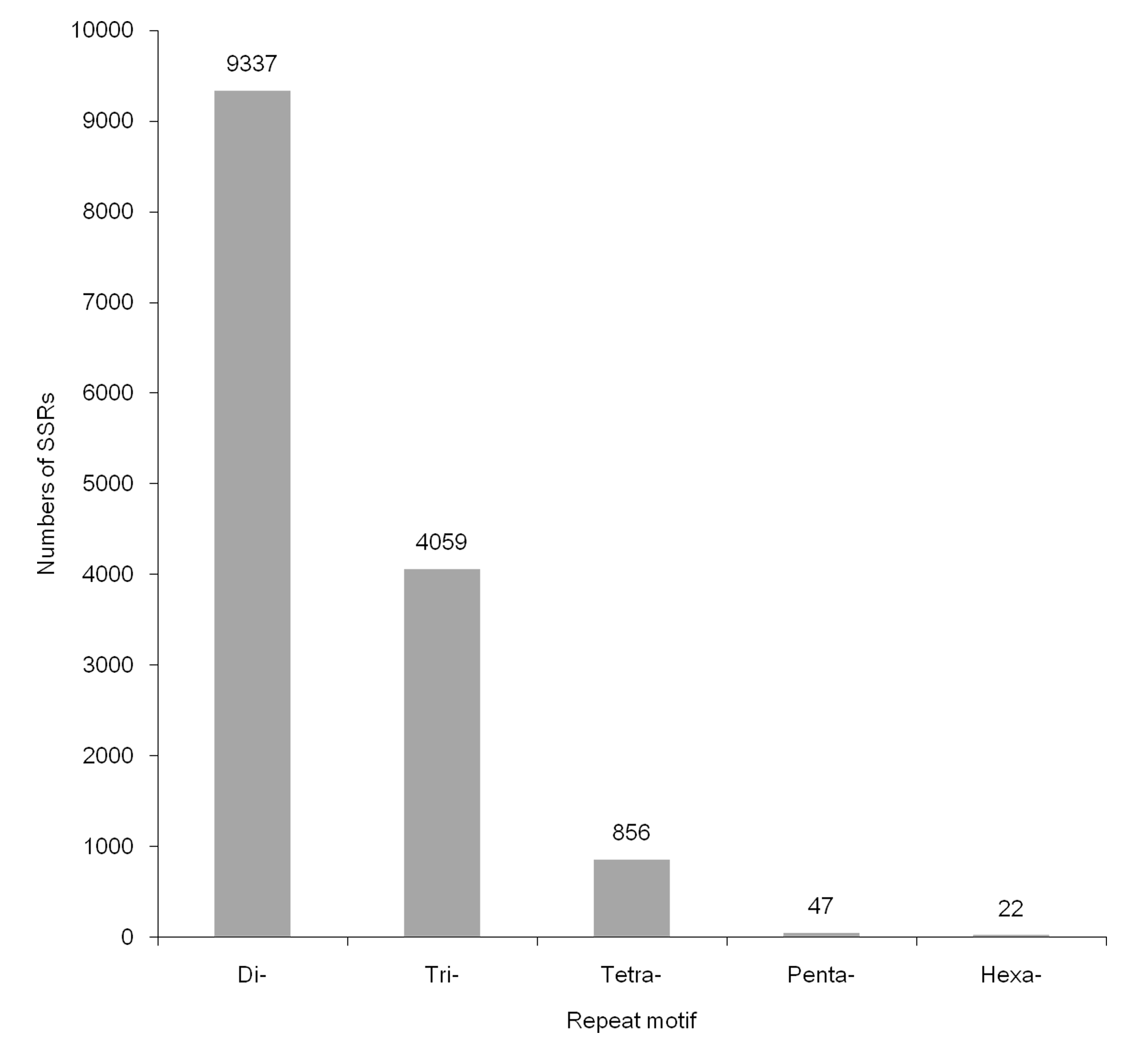

Supplement: S2 Fig — (TIF) [file pone.0185280.s006.tif]
